# Genomic Analyses of a Fungemia Outbreak Caused by Lodderomyces elongisporus in a Neonatal Intensive Care Unit in Delhi, India

**DOI:** 10.1128/mbio.00636-23

**Published:** 2023-04-27

**Authors:** Anamika Yadav, Peeyush Jain, Kusum Jain, Yue Wang, Aditi Singh, Ashutosh Singh, Jianping Xu, Anuradha Chowdhary

**Affiliations:** a Medical Mycology Unit, Department of Microbiology, Vallabhbhai Patel Chest Institute, University of Delhi, Delhi, India; b Department of Zoology, Ramjas College, University of Delhi, Delhi, India; c Department of Paediatrics, Hindu Rao Hospital and NDMC Medical College, Delhi, India; d Department of Biology, McMaster University, Hamilton, Ontario, Canada; e National Reference Laboratory for Antimicrobial Resistance in Fungal Pathogens, Vallabhbhai Patel Chest Institute, University of Delhi, Delhi, India; Institut Pasteur

**Keywords:** *L. elongisporus*, neonate, loss of heterozygosity, phenotypic divergence, ploidy, ascospore, sodium hypochlorite

## Abstract

Lodderomyces elongisporus is a recently emerging yeast pathogen predominantly reported in adult patients who had immunosuppression and/or intravenous access devices. Here, we report a fungemia outbreak caused by *L. elongisporus* in a neonatal intensive care unit (NICU) in Delhi, India, from September 2021 to February 2022. All 10 neonates had low birthweight, and nine of the patients survived after amphotericin B treatment. Whole-genome sequence analyses of the patient isolates as well as those from other sources in India grouped them into two clusters: one cluster consists of isolates exclusively from stored apples and the other cluster includes isolates from patients, clinical environments, and stored apples. All outbreak strains from patients were closely related to each other and showed highly similar heterozygosity patterns across all 11 major scaffolds. While overall very similar, strains from the inanimate environment of the same neonatal intensive care unit showed loss of heterozygosity at scaffold 2 (NW_001813676) compared to the patient strains. Interestingly, evidence for recombination was found in all samples. All clinical strains were susceptible to 10 tested antifungal drugs, and comparisons with strains with high fluconazole MICs derived from the surface of stored apples revealed significant genome divergence between the clinical and apple surface strains, including 119 nonsynonymous single nucleotide polymorphisms (SNPs) in 24 triazole resistance-related genes previously found in other *Candida* spp. Together, our results indicate significant diversity, recombination, and persistence in the hospital setting and a high rate of evolution in this emerging yeast pathogen.

## INTRODUCTION

Invasive fungal infections in neonatal intensive care units (NICUs) have increased significantly worldwide, representing an important infective complication in hospitalized patients. Among hospital-born infants, hospital-acquired infections account for an estimated 4% to 56% of all deaths in the neonatal period, depending on the study population and geographical area (1, 2). Although nosocomial bloodstream infections (BSIs) by bacteria are still more frequent, the frequency of fungal sepsis caused by species of *Candida* and other fungi is increasingly recognized as an important cause of morbidity and mortality in neonatal intensive care units (NICUs) (3–5). In the last 2 decades, several outbreaks of nosocomial BSIs due to rare and emerging yeasts have been reported worldwide, stressing the importance of early detection and timely interventions to prevent and control outbreaks (6–9). Lodderomyces elongisporus is an uncommon cause of fungemia, predominantly reported in adult patients who had immunosuppression and/or intravenous access devices (10–12). This yeast was considered a teleomorph of Candida parapsilosis until sequencing of the 18S rRNA gene differentiated it as a unique species (13). Here, we report an outbreak of fungemia in a NICU involving 10 neonates in a multispecialty hospital in Delhi, India. We used whole-genome sequencing to assess the genetic diversity and relationships among strains obtained from the neonates as well as from the inanimate environment of the hospitals and stored apples from northern India (14).

## RESULTS

### Outbreak investigations.

From September 2021 to February 2022, *L. elongisporus* fungemia occurred in 10 neonates in the 24-bed-capacity NICU for hospital-born neonates of a multispecialty hospital in Delhi, India. Cases of bloodstream infections (BSIs) with this yeast were defined as the isolation of *L. elongisporus* from blood culture of peripheral or central vein blood samples in patients with signs and symptoms of sepsis.

The first case of fungal sepsis occurred in a preterm infant with very low birthweight (<1,500 g) who developed thrombocytopenia and signs of sepsis. The neonate received vancomycin, and meropenem along with fluconazole (FLU) (loading dose of 12 mg/kg of body weight followed by 6 mg/kg of body weight). The patient’s blood culture was positive for the yeast on day 9 after birth. The yeast was identified as *L. elongisporus*, FLU administration was stopped, and liposomal amphotericin B (AMB) was started at a dose of 1 mg/kg of body weight intravenously for 14 days. Overall, 10 cases were identified in neonates hospitalized in the NICU during the period of 6 months. The first four cases (cases 1 to 4) occurred within 2 weeks in September 2021. After the second case of *L. elongisporus* was identified, infection prevention practices were evaluated with the health care workers to mitigate further transmission of the yeast. The quality and frequency of hand hygiene and environmental cleaning were emphasized to the health care staff. Further, two NICU rooms that accommodated *L. elongisporus*-infected neonates were vacated for enhanced cleaning of the rooms. Meanwhile, the neonates were kept in the other designated room in the NICU until the cleaning procedures were satisfactorily completed, which was followed by shifting the neonates to the previous rooms. Subsequently, a reduction in the number of cases was observed, resulting in one case each of fungemia due to *L. elongisporus* in the next 2 months. However, between 31 January and 21 February 2022, a sudden clustering of four cases occurred, and at this point environmental sampling of two sites, i.e., the railing of the neonate open care warmer and its temperature control panel, yielded *L. elongisporus*. Notably, both environmental sites colonized by *L. elongisporus* were cocolonized by C. parapsilosis. Hand swabs of health care workers were found to be negative for any yeast species. Further outbreak investigations were accompanied by strict infection prevention and control practices. Specifically, hand hygiene was practiced before and after each patient contact and before donning or doffing personal protection equipment, and sterilization and daily disinfection were performed for personal protection equipment. The outbreak was contained, and until now (February 2023), no new case of *L. elongisporus* fungemia has been detected in the NICU.

### Clinical details.

Clinical data were extracted from medical records, and the details are described in Table 1. Age at onset of fungemia ranged from 7 to 35 days. All neonates presented a nonspecific sign as increased frequency of apnea episodes; thrombocytopenia (platelet counts of <100 × 10^9^/L) was the early laboratory finding in six neonates. The mean gestational age was 32.4 weeks, and the mean birthweight was 1.1 kg. The risk factors included preterm birth and low/very low birthweight. All neonates were treated with AMB and improved and were discharged, except one who died.

**TABLE 1:** Clinical details of 10 neonates with *Lodderomyces elongisporus* fungemia in a neonatal intensive care unit, India^*a*^

Patient	Isolate ID	DOB/DOA (day-mo-yr)	Date of blood culture (day-mo-yr)	Clinical details	Outcome	Antifungal therapy
1	906-P-21	07-09-2021	16-09-2021	PT, VLBW, thrombocytopenia	Survived	FLU and AMB
2	950-P-21	02-09-2021	24-09-2021	PT, IUGR, thrombocytopenia	Survived	FLU and AMB
3	963-P-21	18-09-2021	28-09-2021	PT, VLBW, severe asphyxia	Survived	FLU and AMB
	979-P-21-S62		30-09-2021			
	979-P-21-S87		19-10-2021			
	1023-P-21		28-10-2021			
4	972-P-21	17-09-2021	30-09-2021	PT, ELBW, thrombocytopenia, hypoglycemia	Survived	FLU and AMB
5	1003-P-21	24-09-2021	12-10-2021	Term, hypoglycemia, thrombocytopenia	Survived	FLU and AMB
6	1107-P-21	01-11-2021	16-11-2021	LBW, PT, thrombocytopenia	Survived	FLU and AMB
7	89-P-22	2-01-2022	31-01-2022	VLBW, PT	Died	FLU and AMB
8	144-P-22	01-02-2022	07-02-2022	Term, LBW, sepsis, thrombocytopenia	Survived	FLU and AMB
9	147-P-22	03-02-2022	09-02-2022	VLBW, severe asphyxia at birth	Survived	FLU and AMB
10	160-P-22	12-02-2022	21-02-2022	VLBW, PT	Survived	FLU and AMB

a ID, identifier; DOA, date of admission; DOB, date of birth; VLBW, very low birthweight; IUGR, intrauterine growth restriction; PT, preterm; ELBW, extremely low birthweight; LBW, low birthweight; FLU, fluconazole; AMB; amphotericin B.

### Strain identification.

A total of 13 bloodstream strains from 10 neonates, including four strains from four serial blood culture specimens from a single case (obtained at 0, 2, 21, and 30 days), were identified as *L. elongisporus* by matrix-assisted laser desorption ionization–time of flight mass spectrometry (MALDI-TOF MS) and confirmed by sequencing of internal transcribed spacer (ITS) regions of the ribosomal DNA (rDNA). All *L. elongisporus* strains on CHROMagar *Candida* medium showed green/blue color at 37°C after 48 h of incubation. Also, the Vitek-2 (bioMérieux, Marcy l’Etoile, France) commercial identification system misidentified *L. elongisporus* as C. parapsilosis with 91 to 96% probability.

### Antifungal susceptibility testing (AFST).

We performed AFST of all *L. elongisporus* strains (13 bloodstream isolates and 2 inanimate-environment strains) from the present outbreak along with three inanimate-environment strains from two other hospitals in Delhi. All strains exhibited low MICs of azoles by the CLSI broth microdilution method (M27-A3) (15). Among azoles, FLU (MIC range, 0.25 to 1 mg/L), voriconazole (VRC; MIC range, <0.03 to 0.06 mg/L), and itraconazole (ITC; MIC range, 0.03 to 0.25 mg/L) showed low MICs. Also, low MICs were observed for AMB (0.125 to 0.5 mg/L), anidulafungin (AFG; <0.015 to 0.5 mg/L), micafungin (MFG; <0.015 to 0.125 mg/L), and 5-flucytosine (5-FC; 0.06 to 0.25 mg/L) (Table 2). We also compared AFST data of 13 *L. elongisporus* strains obtained previously from the surface of stored apples with those of the strains obtained in this study (14). Interestingly, the *L. elongisporus* strains recovered from the surface of stored apples showed 2- to 8-fold-higher fluconazole MICs (MIC range, 2 to 4 mg/L) than the clinical and inanimate-environment strains of *L*. *elongisporus* obtained in the present outbreak.

**TABLE 2:** MIC distribution of *Lodderomyces elongisporus* strains isolated in the present outbreak including clinical and inanimate sources along with strains obtained from other sources against 10 antifungal drugs using CLSI-broth microdilution method

*L. elongisporus* (no. of isolates)	MIC range (mg/L)^*a*^								
	FLU	ITC	VRC	ISA	POS	AMB	MFG	AFG	5-FC
Clinical, bloodstream isolates (*n* = 13)	0.25–1	0.03–0.25	<0.03–0.06	<0.015	<0.015–0.125	0.125–0.5	<0.015–0.125	<0.015–0.5	0.06–0.25
Inanimate environment, NICU (neonate warmer; *n* = 2)	0.5	0.03–0.06	<0.03	<0.015	<0.015	0.25	<0.015	<0.015–0.5	0.06
Inanimate environment of two hospitals, Delhi (floor; *n* = 3)^*b*^	0.5	0.03–0.06	<0.03	<0.015	<0.015	0.25	<0.015	<0.015–0.5	0.06
Strains from surfaces of apples (*n* = 13)^*c*^	2–4	0.25	0.03–0.12	<0.015	0.06–0.5	0.01–0.5	0.01	0.01–0.5	0.06–0.12

a FLU, fluconazole; ITC, itraconazole; VRC, voriconazole; ISA, isavuconazole; POS, posaconazole; AMB, amphotericin B; MFG, micafungin; AFG, anidulafungin; 5FC, flucytosine.

b Two strains obtained in the previous study (16).

c Strains obtained in the previous study (14).

### Genome analysis.

**FIG 1:**
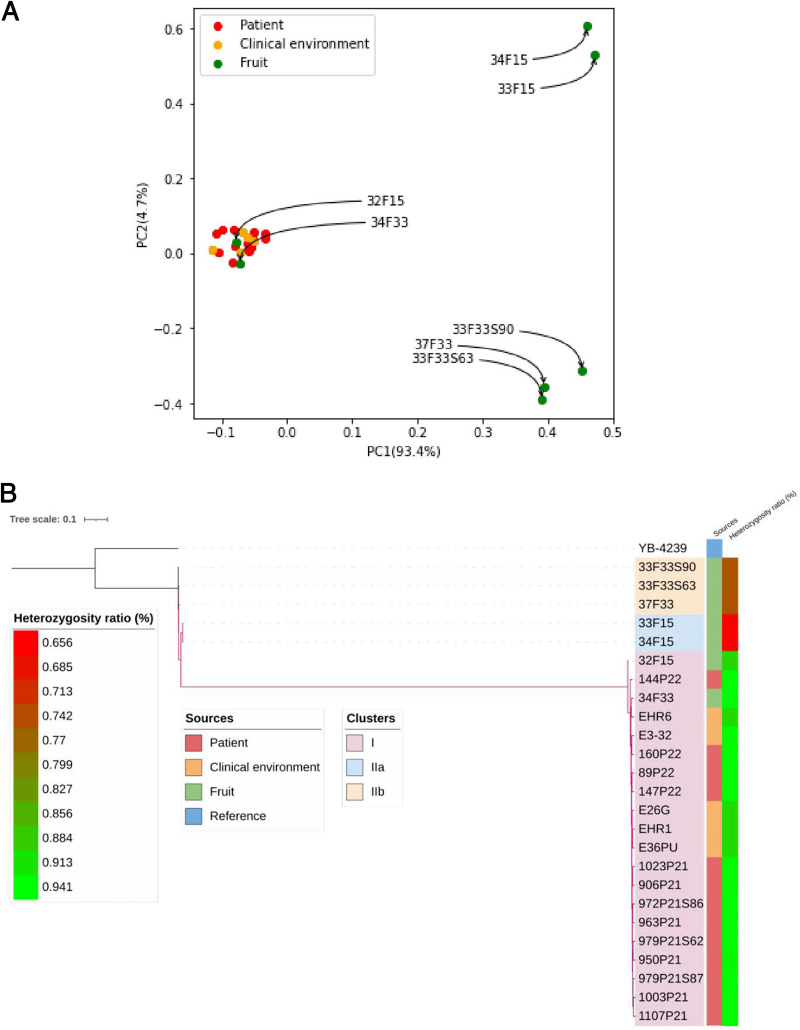
(A) Principal components unveil population structure of *Lodderomyces elongisporus* samples from three sources, i.e., clinical (patient), inanimate environment (clinical environment), and fruits (apple surface). (B) Genetic relatedness among 25 isolates and the reference strain inferred based on homozygous SNPs. Branches with bootstrap support over 0.95 are highlighted in purple. The inner color strips indicate the sources of the isolates. The outer color gradients display the heterozygosity ratio of each sample calculated by the number of heterozygous sites relative to reference genome size.

The genomes of 13 clinical strains and two inanimate-environment strains from the present outbreak were analyzed along with the seven *L*. *elongisporus* strains isolated from the surface of stored apples and three environmental strains obtained from the floor of two other hospitals (14, 16). The reference strain YB-4239 genome was included for single nucleotide polymorphism (SNP) calling and phylogenetic analysis (17). The isolates belonged to two clusters, cluster I and cluster II. All clinical and inanimate-hospital-environment strains of three hospitals were closely related (3,260- to 4,581-SNP difference) and comprised a tight genotype cluster I. Interestingly, two strains from individual apple surfaces (VPCI/34/F33/2020, and VPCI/32/F15/2020) clustered with clinical and inanimate-environment strains in cluster I (Fig. 1A). The remaining five strains obtained from surfaces of stored apples formed cluster II, comprising two subclusters (cluster IIa, *n* = 2; cluster IIb, *n* = 3; 4,984- to 5,387-SNP difference between subclusters IIa and IIb). Overall, the cluster II isolates from the surfaces of apples were genotypically divergent from cluster I strains, with 176,721 to 177,579 SNPs separating them (Fig. 1A and B). All *L. elongisporus* strains in cluster I (clinical, inanimate environment, and two fruit strains) had identical genotypes at the 24 drug-resistance-related genes (see Table S1 in the supplemental material). Interestingly, strains from surfaces of apples (cluster II) which were genetically distinct from the clinical and inanimate-environment strains (cluster I) had a total of 119 nonsynonymous substitutions in 24 triazole-resistance-related genes previously identified in *Candida* spp. (Table S1), including three mutations in *ERG11* (Met15Leu, Ser183Thr, and Asn363Ile). Further, strains in cluster II from surfaces of apples which were genetically distinct from clinical and inanimate-environment strains showed partial or complete loss of heterozygosity (LOH) in scaffolds 1 (NW_001813675) and 6 (NW_001813680) (Fig. S1). Similarly, environmental strains obtained from the neonate warmer (*n* = 2; VPCI/E/HR6/2022 and VPCI/E/HR1/2022) and inanimate-environment strains (*n* = 2; VPCI/E/28G/2020 and VPCI/E/36PU/2020) from the floor of other hospitals also showed LOH in scaffold 2 (NW_001813676).

10.1128/mbio.00636-23.1 FIG S1 Loss of heterozygosity pattern of 25 *Lodderomyces elongisporus* strains including 13 clinical strains and two inanimate-environment strains from the present outbreak, seven strains isolated from the surface of stored apples, and three environmental strains obtained from the floor of two other hospitals over the 11 scaffolds. Samples are ordered based on isolation sources. Samples from patients, clinical environment, and fruit are marked by red, orange, and green labels. Purple and yellow blocks indicate heterozygous and homozygous regions, respectively. Download FIG S1, DOCX file, 0.5 MB. Copyright © 2023 Yadav et al. 2023 Yadav et al. https://creativecommons.org/licenses/by/4.0/ This content is distributed under the terms of the Creative Commons Attribution 4.0 International license.

10.1128/mbio.00636-23.6 TABLE S1 SNP difference at 24 antifungal resistance-related genes between 13 clinical isolates of *Lodderomyces elongisporus* along with five isolates from inanimate environment of three different hospitals and seven isolates from surfaces of apples. Download Table S1, XLSX file, 0.2 MB. Copyright © 2023 Yadav et al. 2023 Yadav et al. https://creativecommons.org/licenses/by/4.0/ This content is distributed under the terms of the Creative Commons Attribution 4.0 International license.

### Morphological characterization of *Lodderomyces elongisporus*.

On acetate ascospore agar, all bloodstream isolates of *L. elongisporus* developed abundant ascospores within 2 weeks of incubation at 37°C (Fig. S2). Also, a few strains from surfaces of apples (*n* = 3, VPCI/33F33/2020, VPCI/34F33/2020, and VPCI/37F33/2020) showed abundant ascospore formation after 3 weeks of incubation. Interestingly, one of the fruit strains (VPCI/34F33/2020) producing ascospores was genetically related to clinical strains in cluster I. In contrast, only one strain from the inanimate environment of the NICU developed a few (1 to 2/slide) ascospores after 3 weeks of incubation.

10.1128/mbio.00636-23.2 FIG S2 Ascospore formation in a clinical isolate (VPCI/160/P/2022) on ascospore agar containing 0.1% sodium acetate stained with lactophenol cotton blue at ×40 magnification showing one or two ascospores (white arrow) enclosed in an ascus. Download FIG S2, DOCX file, 5.1 MB. Copyright © 2023 Yadav et al. 2023 Yadav et al. https://creativecommons.org/licenses/by/4.0/ This content is distributed under the terms of the Creative Commons Attribution 4.0 International license.

### Determination of ploidy in *Lodderomyces elongisporus*.

We examined the genomic DNA content of 13 *L. elongisporus* isolates including two randomly selected clinical (VPCI/147/P/2022 and VPCI/160/P/2022) strains, all five strains from the inanimate environment of hospitals, and six strains recovered from the surface of apples using fluorescence-activated cell sorting (FACS) assays. Overall, most *L. elongisporus* strains had 2n DNA content (diploid), similar to the Candida albicans reference strain ATCC 90028, except two strains obtained from the open warmer of the NICU. These two strains recovered from the inanimate environment of the present outbreak showed distinct genomic DNA (aneuploid), between haploid and diploid (Fig. S3).

10.1128/mbio.00636-23.3 FIG S3 Histograms representing DNA content or cell cycle profile obtained by FACS. For comparison, diploid C. albicans ATCC 90038 was used as a reference strain. The *x* axis represents nuclear fluorescence, and the *y* axis represents cell number. Download FIG S3, DOCX file, 0.2 MB. Copyright © 2023 Yadav et al. 2023 Yadav et al. https://creativecommons.org/licenses/by/4.0/ This content is distributed under the terms of the Creative Commons Attribution 4.0 International license.

### Susceptibility to disinfectants.

We investigated the susceptibility pattern of four classes of disinfectants against all strains including low-level disinfectant (absolute ethanol) and high-level disinfectants, i.e., glutaraldehyde, fifth-generation quaternary ammonium compound, and sodium hypochlorite. We compared the activities of disinfectant on clinical strains, strains from the inanimate hospital environment, and strains obtained from the apple surfaces. Overall, all 25 *L*. *elongisporus* isolates showed low MICs against all disinfectants tested except sodium hypochlorite. Specifically, high MIC values (MIC value of ≥1%) were observed for sodium hypochlorite against the majority of clinical strains (85%), all inanimate-environment strains from hospitals, and a single fruit strain which was genetically related to clinical strains. Notably, for sodium hypochlorite, all inanimate-environment strains and 50% of clinical strains showed 2- to 4-fold-higher MICs (2% to 4%) than the recommended concentration (1%, equivalent 1,000 ppm) of sodium hypochlorite used in clinical practices for disinfection (18). In contrast, five fruit strains distinct from the outbreak cluster (cluster II) showed low sodium hypochlorite MICs (0.06% to 0.5%).

### Time-kill kinetics.

As *L. elongisporus* strains exhibited high MIC values for sodium hypochlorite, we investigated the activity of sodium hypochlorite on yeast cells, using a time-kill curve assay. The time-kill kinetics profile of clinical and inanimate strains against 1% sodium hypochlorite showed that both types of strains had lower susceptibility to sodium hypochlorite than did strains from apple surfaces (Fig. S4). Further, after 8 h, inanimate-environment and clinical strains from cluster I that exhibited high MICs for sodium hypochlorite (MIC value of >1%) showed prominent growth in the presence of 1% sodium hypochlorite, with growths not significantly different (<2-fold difference; *P* > 0.05) from that of control (absence of sodium hypochlorite). In contrast, after 8 h, fruit strains treated with sodium hypochlorite showed prominent growth inhibition (>2-fold difference; *P* < 0.05) compared with untreated cells.

10.1128/mbio.00636-23.4 FIG S4 The graph illustrates the time-kill curve (optical density versus time plot) of three *Lodderomyces elongisporus* strains from clinical (VPCI/160/P/2022), neonate warmer (inanimate environment, VPCI/E/HR6/2022), and apple surface (VPCI/32F15/2020) sources in the presence of 1% (vol/vol) sodium hypochlorite. The untreated inoculum was used as a control. Download FIG S4, DOCX file, 0.07 MB. Copyright © 2023 Yadav et al. 2023 Yadav et al. https://creativecommons.org/licenses/by/4.0/ This content is distributed under the terms of the Creative Commons Attribution 4.0 International license.

Further, the viability of all the three strains in the presence of 1% sodium hypochlorite was examined by spreading the inoculum on Sabouraud dextrose agar (SDA) plates after 2-h intervals up to 24 h of the incubation period at 37°C. Among the three isolates, the inanimate-environment strain grew luxuriantly in the presence of 1% sodium hypochlorite within 2 h. However, the strain from the surface of fruit was killed in the presence of 1% sodium hypochlorite after 2 h (Fig. S5).

10.1128/mbio.00636-23.5 FIG S5 Representation of viable cells on culture plates by in-use testing in the presence of 1% sodium hypochlorite. Time of incubation is depicted horizontally. Panels A (VPCI/160/P/2022; clinical), B (VPCI/32F15/2020; from apple surface), and C (inanimate environment VPCI/E/HR6/2022; neonate warmer) represent three *Lodderomyces elongisporus* strains in the absence of sodium hypochlorite. Panels D, E, and F represent the three *L. elongisporus* strains in the presence of 1% sodium hypochlorite in similar order. Download FIG S5, DOCX file, 0.4 MB. Copyright © 2023 Yadav et al. 2023 Yadav et al. https://creativecommons.org/licenses/by/4.0/ This content is distributed under the terms of the Creative Commons Attribution 4.0 International license.

### SEM of *Lodderomyces elongisporus* after 1% sodium hypochlorite treatment.

**FIG 2:**
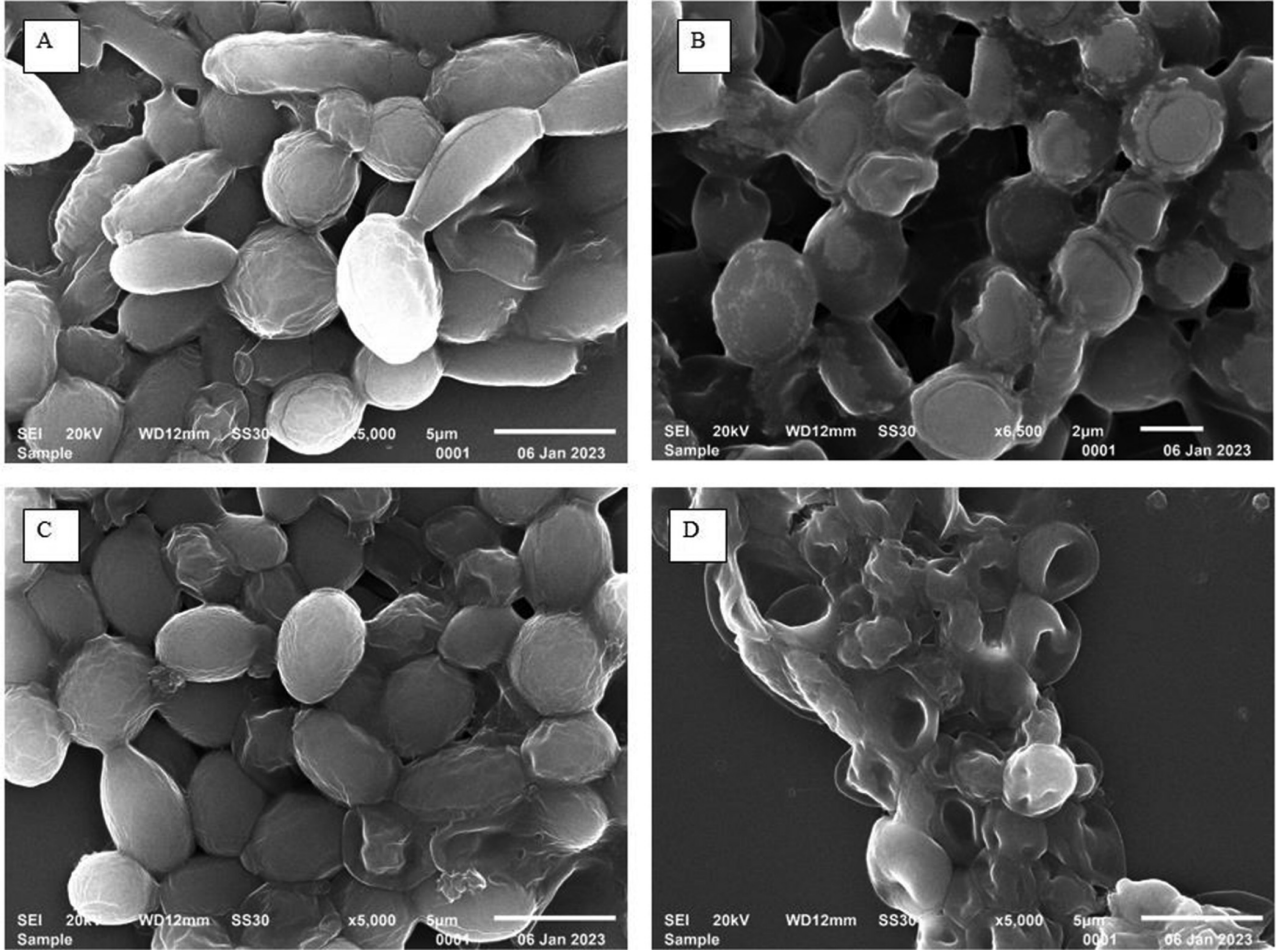
Scanning electron micrograph of *Lodderomyces elongisporus*. (A) VPCI/160/P/2022 clinical strain without 1% sodium hypochlorite treatment. (B) VPCI/160/P/2022 clinical strain with 1% sodium hypochlorite. (C) SEM images of VPCI/E/HR6/2022 inanimate clinical environmental strain without 1% sodium hypochlorite treatment. (D) VPCI/E/HR6/2022 inanimate clinical environmental strain with 1% sodium hypochlorite.

During adaptation to different microenvironments, morphological features of yeast cells may change. Therefore, we investigated the morphological changes among the clinical and inanimate-environment strains from hospitals in the presence of disinfectant using scanning electron microscopy (SEM). Scanning images of cells were captured before and after treatment with 1% sodium hypochlorite. Before treatment, the clinical strain showed ovoid budding yeast cells along with tube-like blastoconidia whereas cells of the inanimate-environment strain (NICU warmer) showed only spherical, ovoid yeast cells. After treatment of cell inoculum with 1% sodium hypochlorite, the damaging effect was prominent in the inanimate-environment strain compared to the clinical strain. Deep invaginations over cell surfaces were observed in the environmental strain whereas the clinical strain did not exhibit such changes (Fig. 2).

### Mode of reproduction.

*L. elongisporus* is diploid and has been described as sexual and homothallic (self-mating) (17). However, previous studies failed to identify mating-type-like (MTL) genes that were homologous to those in *Candida* spp. in *L. elongisporus*. Here, we used both the nucleotide and amino acid sequences of *MTL* genes in Candida albicans and C. parapsilosis to search for potential matches in our strains. Using TBLASTN searches, a strong significant match (5e^−150^) to MTLa1p of C. parapsilosis was found on scaffold 7 in all our strains as well as in the reference strain but no match was found for MTLa2p in the genomes of any of our sequenced strains or the reference strain. Adjacent to the MTLa1 locus on scaffold 7 of *L. elongisporus*, homologs to several other genes of the C. albicans *MTL*a locus were found, including *RCY1*, *PIK*a, *OBP*a, and *PAP*a. Interestingly, a very weak match (3e^−04^) was found for MTLα2p of C. albicans on scaffold 2 in all *L. elongisporus* strains. However, no match was found for MTLα1p in the genomes of any of our sequenced strains or the reference strain. Together, the results suggest that the *L. elongisporus* strains analyzed here each contained partial sequences of both the *MTL*a and *MTL*α loci found in several other related *Candida* species. Combined with the observation of ascospore formation in several of the strains, the genomic results suggest that *L. elongisporus* is capable of sexual reproduction via homothallism.

In homothallism, a single individual cell can complete sexual reproduction by itself. In haploid organisms, homothallic reproduction results in progeny with genotypes identical to those of parental strains, similar to those in clonal/asexual reproduction. However, in diploid organisms, homothallism can produce recombinant genotypes. To investigate whether there is evidence for recombination, we conducted a four-gamete test for our samples. In this test, assuming no parallel or reverse mutations, the presence of all four genotypes at two loci with two alleles each in the population would be consistent with recombination. Because *L. elongisporus* is a diploid, we used only homozygous SNPs for this test. In addition, only SNPs with the minor-frequency allele present in at least two strains were included for analysis. Our analyses revealed that in the total sample of 25 isolates, 0.96% (out of 16,653,581,253) of the analyzed SNP pairs were consistent with recombination. However, for the clinical environmental population and the patient population of *L. elongisporus*, 59.72% (out of 3,901,821) and 64.76% (out of 51,770,400) of the SNP pairs were consistent with recombination. In contrast, only 0.19% (out of 15,321,513,826) of the SNP pairs in the fruit sample were consistent with recombination. Together, the results suggest that *L. elongisporus* strains were undergoing infrequent sexual recombination in the fruit population but frequent recombination in the patient population and in the hospital environment.

## DISCUSSION

We report the outbreak of *L. elongisporus* in a NICU and confirm the close genetic relationships among strains obtained from the neonates, those from their inanimate environment, and those from the surfaces of apples using whole-genome sequencing. The present outbreak of *L. elongisporus* infection affected primarily preterm neonates with low birthweight. Though overall infrequent, *L. elongisporus* infections have been mainly reported as sporadic cases occurring primarily in adult patients with intravenous drug use and in patients with cardiac-related conditions (10, 12, 19, 20). Invasive fungal infections caused by *L. elongisporus* have been reported from diverse geographical regions including countries in the Middle East and East Asia, as well as from India, Spain, Australia, North America, and Canada (21–23). Recently, *L. elongisporus* bloodstream infections were reported in three infants and five adults in a single center in North India. Among the pediatric cases, underlying diseases included congenital heart disease/atrophic kidney and tracheoesophageal fistula (22). Also, a single case in a pediatric patient with a ventricular septal defect was reported from Delhi, India (23).

*L. elongisporus* was first recovered from citrus concentrate in 1952 and named Saccharomyces elongisporus. This yeast was later renamed *L. elongisporus* by van der Walt and colleagues in 1966 (24). The epidemiologic clustering of *L. elongisporus* in 10 neonates in the span of 6 months suggested transmission of this yeast among the hospitalized neonates. The fact that the railing and the temperature panel of the open care warmer of the *L. elongisporus*-positive neonate also harbored this yeast suggested a potential link between the environmental strains and the neonates. Indeed, whole-genome sequence analyses revealed the overall high genome sequence similarity between strains from the inanimate environment of the hospital and those infecting the neonates. Further, two strains obtained from surfaces of apples also showed genetic relatedness with the patient and inanimate-clinical-environment sources in hospitals, suggesting *L. elongisporus* strains could be exchanged among patients and hospital environments and outside the clinical environments. Previously, strains of Candida auris isolated from surfaces of apples in Delhi, India, were found to be genetically linked to clinical strains, suggesting fruits as a possible source of transmission of the yeast and vice versa (14).

Surprisingly, we found evidence for recombination in all three ecological samples, especially those from the inanimate clinical environment and neonates. Indeed, the high genetic similarity among strains within both cluster I and cluster II initially suggested evidence of clonal expansion of one or a few strains in India. The low rates of SNP pairs showing evidence of recombination within both the total sample and the fruit sample were due to genetic structuring within these two samples where two divergent clusters were included in these analyses, contributing to a high number of SNPs being analyzed. The inclusion of divergent populations in the same four-gamete test would vastly increase the number of SNP pairs in the analyses (i.e., increased denominator) and, if there were no outcrossing and recombination between the divergent populations, would reduce the percentages of SNP pairs with evidence of recombination. Our observed results further support the genetic distinctiveness of clusters I and II.

The genome-wide heterozygosity patterns showed a unique loss of heterozygosity (LOH) for a large part of scaffold 2 (NW_001813676) in the strain obtained from the open warmer in the NICU. Such an observed LOH could be derived from sexual, asexual, or parasexual reproduction (25). In addition, neonatal environment strains exhibited aneuploidy. Notably, inanimate-environment strains exhibited resistance to a commonly used surface disinfectant, i.e., sodium hypochlorite (MIC, >1% or >1,000 ppm of active chlorine), used for disinfecting soiled surfaces. In the present study, the broth microdilution method employed for disinfectant testing used RPMI growth medium, which may consume some amounts of available chlorine. However, consistent results were obtained with repeated testing which also correlated with the time-kill assay. In health care settings various guidelines recommend 1,000 ppm of active chlorine for routine disinfection of patient care areas and equipment and higher concentration of 10,000 ppm for terminal cleaning against *Candida* species, including Candida auris (26–28). Also, a similar concentration of sodium hypochlorite is suggested for surface disinfection against multidrug-resistant bacteria like Acinetobacter baumannii and Klebsiella pneumoniae (29, 30). Exposure to antifungal drugs is known to create aneuploidy or genome instability in a diversity of fungal pathogens, causing drug resistance/tolerance (31). Indeed, disinfectants and antifungal drug exposure have likely been a driving force for selection or faster evolution of the inanimate environmental and clinical strains in this study.

Diploid yeasts such as C. albicans and Candida tropicalis often are heterozygous for many genetic variants across the genome (32). Nevertheless, studies have pointed out that regions of the genome can frequently become homozygous through sexual, parasexual, and asexual processes (24). Loss of heterozygosity events can be induced by stress; notably, several stressors including exposure to heat, an oxidizing agent such as hydrogen peroxide, and fluconazole in C. albicans and Cryptococcus neoformans serotype allodiploid (AD) hybrids increased LOH even at doses that did not inhibit growth (33, 34). Typically, in these cases, the LOH events were followed by the duplication of the remaining chromosome/chromosomal segments to restore diploidy. It is tempting to speculate that the environmental strains of *L. elongisporus* isolated in the present study exhibited LOH probably due to the presence of several stressors in the hospital environment, such as enhanced use of disinfectants. Regardless, the seemingly rapid genome change in the hospital environment suggests that *L. elongisporus* is capable of fast evolution and adaptation in stressed environments. Indeed, *L. elongisporus* strains have been recovered from various environmental sources including fresh fruit, fruit concentrates, soft drinks, and insects (10, 35, 36).

We found a range of unique SNPs within each of the strains, including those from patients. Some of these SNPs in strains from patients were likely *de novo* mutations accumulated during their asexual reproduction. Interestingly, our genome comparisons revealed a large number of mutations in genes involved in DNA repair, including both missense mutations and four base substitutions that caused predicted “stop_gained”/“stop_lost”/“start_lost”/“stop_gained” functions in four genes annotated to be involved in DNA repair in all our clinical strains compared to the reference strain NRRL YB-4239. Specifically, those included (i) a translation “stop_gained” A→T mutation at nucleotide position 516372 of scaffold NW_001813679 of gene LELG_04803 annotated as involved in “DNA repair” where all clinical strains were homozygous for the mutant nucleotide T at this position, (ii) a translation “stop_lost” A→T mutation at nucleotide position 391028 of scaffold NW_001813680 of gene LELG_04316 annotated as involved in “DNA repair” where all clinical strains were heterozygous A/T at this position, (iii) a translation “start_lost” T→C mutation at nucleotide position 901485 of scaffold NW_001813680 of gene LELG_04499 annotated as involved in “nucleotide excision repair” where all clinical strains were homozygous for mutant allele C/C at this position, and (iv) a translation “stop_gained” C→T mutation at nucleotide position of 185369 of scaffold NW_001813681 of gene LELG_03735 annotated as involved in “nucleotide excision repair” where all clinical strains were heterozygous C/T at this position (see Table S1 in the supplemental material). Together, these mutations could have contributed to the high number of unique SNPs observed for each strain.

In the present outbreak, infection was likely transmitted among neonates by contact with health care workers and fomites. Suboptimal adherence to infection prevention and control practices is usually a major contributing factor to outbreaks, which probably was the case in the present outbreak. Of neonates infected with *L. elongisporus*, the majority had very low birthweights and were born preterm. Prematurity and low birthweight are well-recognized risk factors for candidemia. All the neonates were empirically treated with fluconazole, and amphotericin B was instituted after the confirmation of *L. elongisporus*. In fact, all the bloodstream strains exhibited susceptibility to azoles, amphotericin B, and echinocandins. Interestingly, strains obtained from the natural environment, i.e., from the surfaces of apples (*n* = 7), showed 2- to 8-fold-increased MICs of azoles compared to clinical strains. Further, the strains obtained from surfaces of apples showed alterations in the azole target genes such as *TAC1*B, *CDR1*, and *MDR1*. These findings suggest that continued exposure to azoles in the environment may select azole-resistant strains of *L. elongisporus*. Finally, the multifaceted investigation of the *L. elongisporus* outbreak in the present study suggests that this yeast is among a growing list of fungi capable of causing severe infections among a diversity of human hosts (37). The genetic mechanisms underlying their adaptations to humans and hospital and natural environments warrant in-depth investigations and measures to contain their spread and persistence in clinics.

## MATERIALS AND METHODS

### Microbiological investigation.

Blood culture specimens obtained from all neonates flagged positive in the BacT/Alert 3D system (bioMérieux, Inc., Durham, NC). Subculture of the positive blood culture bottles was undertaken on Sabouraud dextrose agar (SDA), and the colony characteristics of *L. elongisporus* on SDA and CHROMagar *Candida* medium (Becton, Dickinson, Baltimore, MD, USA) were recorded at 37°C. Further, biochemical profiling of isolates using the Vitek-2 (bioMérieux, Marcy l’Etoile, France) commercial identification system was evaluated (38). The identification of isolates to species level was done by matrix-assisted laser desorption ionization–time of flight mass spectrometry (MALDI-TOF MS; Bruker Biotyper OC version 3.1; Daltonics, Bremen, Germany) (≥2 score) and confirmed by sequencing of internal transcribed spacer (ITS) regions of the ribosomal DNA (rDNA) (38).

### Environmental sampling.

A total of 80 swabs (Himedia, Mumbai, India) were collected from the surrounding inanimate environments where *L. elongisporus*-positive neonates resided within the hospital. Environment samples were collected at the end of February 2022, after the sudden surge of four fungemia cases in the neonatal intensive care unit (NICU). We collected swab samples from all neonatal open care warmers including their surfaces, railings, monitoring panels attached to warmers, such as temperature display panels, pulse oximeter probes, bedsheets, and respiratory care equipment. Also, trays containing disposable supplies, adjacent floor trolleys, water sink, taps, the hand dryer, doorknobs, and weighing machines were thoroughly swabbed (16). Simultaneously, swabs from fingertips and finger webs of health care workers at the neonate ICU were also collected to check for colonization (39).

### AFST.

A total of 31 *L. elongisporus* isolates recovered from clinical specimens (*n* = 13 BSIs) and inanimate-environment isolates (*n* = 2) from the NICU, from the floor of two other hospitals (*n* = 3), and from the surfaces of stored apples (*n* = 13) were subjected to antifungal susceptibility testing (AFST) by the CLSI broth microdilution method following method M27-A3 (15). The isolates were tested against 10 antifungal drugs of three classes, i.e., azoles, echinocandins, and amphotericin B, as detailed in Table 2. The antifungals tested were fluconazole (FLU; Sigma, St. Louis, MO, USA), itraconazole (ITC; Lee Pharma, Hyderabad, India), voriconazole (VRC; Pfizer, Groton, CT, USA), posaconazole (POS; Merck, Whitehouse Station, NJ, USA), isavuconazole (ISA; Basilea Pharmaceutical, Basel, Switzerland), 5-flucytosine (5-FC; Sigma), caspofungin (CFG; Sigma), micafungin (MFG; Sigma), anidulafungin (AFG; Sigma), and amphotericin B (AMB; Sigma). The drugs were tested for 10 (2-fold) dilutions, and the drug concentration ranges were as follows: FLU, 0.25 to 128 mg/L; ITC, VRC, and AMB, 0.03 to 16 mg/L; POS, ISA, AFG, MFG, and CFG, 0.015 to 8 mg/L; 5-FC, 0.125 to 64 mg/L. Candida krusei strain ATCC 6258 and Candida parapsilosis strain ATCC 22019 were used as quality control strains.

### Whole-genome sequencing and phylogenetic analysis.

We sequenced whole genomes from each selected isolate (*n* = 13 clinical isolates, *n* = 2 environmental isolates) obtained from the present outbreak. In addition, we selected 10 *L. elongisporus* isolates, seven from the surface of stored apples and three from the floor of two other hospitals in Delhi, using a NextSeq sequencer. Whole-genome sequence libraries were prepared using the NEBNext Ultra II DNA FS kit (New England Biolabs, Ipswich, MA, USA). Whole-genome sequencing data of 25 Indian samples were first filtered for low-quality reads and adapters using Trimmomatic v0.39 (40). Then, clean reads were aligned with the genome of *L. elongisporus* strain YB-4239 (GCF_000149685.1_ASM14968v1_genomic.fna) using BWA-MEM v0.7.17 (41). Postalignment processing was performed using SAMtools v1.13 and Picard tools v2.26.3 (http://broadinstitute.github.io/picard/). Variant identification was conducted using GATK v4.2 and GATK tools HaplotypeCaller, CombineGVCFs, GenotypeGVCFs, SelectVariants, and Variant Filtration (42). Single nucleotide polymorphisms (SNPs) were selected by removing SNP loci that failed to meet the following hard thresholds “QualByDepth (QD) < 2.0 ‖ FisherStrand (FS) > 60.0 ‖ RMSMappingQuality (MQ) < 40.0 ‖ StrandOddsRatio (SOR) > 4.0”. The specific filters are as follows: QD: the variant confidence normalized by unfiltered depth of variant samples; FS: strand bias estimated using Fisher’s exact test; MQ: root mean square of the mapping quality of reads across all samples; SOR: strand bias estimated by the symmetric odds ratio test. Phylogenetic analysis was conducted on homozygous loci containing SNPs in at least one sample (43). Maximum likelihood phylogeny was inferred with the GTR+CAT model based on 244,443 homozygous loci using FastTree v 2.1.11 (44). We deposited FASTQ files for all isolates at GenBank under BioProject no. PRJNA889843.

### Four-gamete test for evidence of recombination.

To infer whether recombination has occurred in our samples, four-gamete tests were conducted, following procedures described in reference 40. Specifically, strains were first grouped based on isolation sources, i.e., clinical environment (5 isolates), patients (13 isolates), fruit (7 isolates), and the total population (all 25 isolates). Then, pairs of homozygous SNP loci with the minor allele present in at least 2 strains of each group were compared. Here, for any two SNP sites containing both the reference allele (0) and an alternative allele (1), if all four possible combinations, i.e., 00, 01, 10, and 11, were identified, we considered the SNP pair consistent with recombination.

### Morphological characterization of *Lodderomyces elongisporus*.

To check any morphological difference between *L. elongisporus* strains on the basis of their source of isolation, all were subcultured on ascospore agar containing 0.1% sodium acetate and incubated at 37°C for ≥21 days. Cells were monitored under an optical microscope (Nikon H600L; Japan) at ×40 magnification.

### Determination of ploidy of *Lodderomyces elongisporus* strains.

A total of 13 *L. elongisporus* isolates including two clinical strains (VPCI/147/P/2022 and VPCI/160/P/2022), all five strains from the inanimate environment, and six strains recovered from the surface of fruits were subjected to fluorescence-activated cell sorting (FACS) (FACSAria III; BD Biosciences, USA). Candida albicans (ATCC 90028) was used as a reference for the diploid strain. Sample preparation and analysis were done as described previously (14). FlowJo 7.8 software was used to interpret the FACS results.

### Activity of disinfectants against *Lodderomyces elongisporus*.

A disinfectant activity assay was done to compare the activities of disinfectants on clinical strains, strains from the inanimate environment, and strains from the apple surfaces. In this study, four disinfectants were used, which were the intermediate-level disinfectant absolute ethanol (Changshu Hongsheng Fine Chemical, China) and high-level disinfectants glutaraldehyde (2.45% [wt/vol]; Sceptre Medical India, Delhi, India), fifth-generation quaternary ammonium compound (13.6% [wt/vol] Mi-Fog [Faith Microsolution, Delhi, India]), and sodium hypochlorite containing 10% available chlorine (Ebele Cosmeceuticals, India). The broth microdilution method was used for determining MICs of the disinfectants against 25 strains of *L. elongisporus*. All the disinfectants were tested for 10 (2-fold) dilutions, and the drug concentration ranges were 0.06 to 32% for sodium hypochlorite, corresponding to 6 to 3,200 ppm of available chlorine, and 0.19 to 100% for ethanol, quaternary ammonium compound, and glutaraldehyde. MIC endpoints for disinfectants were defined as the lowest concentration that inhibited 100% of the growth as read visually at 24 h of incubation at 37°C.

### Time-kill assay of *Lodderomyces elongisporus* against disinfectant (sodium hypochlorite).

Three isolates including one from each source, i.e., clinical (VPCI/160/P/2022), inanimate environment (VPCI/E/HR6/2022), and apple surface (VPCI/32F15/2020), were grown in SD broth at 37°C for 16 h. Briefly, an inoculum of 1 × 10^8^ cells (optical density at 625 nm [OD_625_] = 0.1) from an overnight culture was inoculated again into SD broth for 3 h. After 3 h of incubation, suspensions were supplemented with 1% sodium hypochlorite (vol/vol; recommended concentration of sodium hypochlorite). An untreated inoculum of yeast cells was used as a control. Further, a 200-μL inoculum of both treated and untreated cells was dispensed into a 96-well microtiter plate. The plate was incubated inside a microplate reader (Infinite 200 Pro; Tecan, Switzerland) with constant agitation at 200 rpm at 37°C. The growth turbidity was measured at a regular interval of 2 h for 24 h at an optical density at 530 nm. The growth curve was plotted against OD_530_ versus time by using GraphPad Prism 4. In addition, in-use testing was performed for the same three isolates (45). An inoculum with an OD_625_ of 0.1 of each isolate was transferred to a 15-mL solution of 1% sodium hypochlorite and incubated at 37°C. The kill rate of *L. elongisporus* strains in the presence of sodium hypochlorite solution was determined by examining the viability of fungal cells after 0-, 2-, 4-, 6-, 8-, and 24-h periods of contact with the 1% sodium hypochlorite solution. To examine the viable cells in suspension, 100 μL of inoculum after every 2 h was taken and subcultured on SDA plates and images of culture plates were captured by a gel imager (Bio-Rad, CA, USA) after 24 h of incubation.

### Scanning electron microscopy of *Lodderomyces elongisporus*.

To evaluate the effect of sodium hypochlorite on the surface of *L. elongisporus* cells, scanning electron microscopy (SEM) was done. One clinical strain (VPCI/160/P/2022) and one strain from the inanimate environment (VPCI/E/HR6/2022) were selected for this purpose. Microbial samples for SEM analysis were prepared as described previously (46). A single colony of both selected strains was subcultured on SDA followed by inoculation onto the yeast extract-peptone-dextrose (YPD) broth incubated at 37°C for 17 h in a shaking incubator at 200 rpm. An inoculum with 1 × 10^8^ cells (OD_600_ = 0.1) from overnight culture was transferred in YPD broth. A prepared volume of cell suspension was further divided into two equal volumes. One was supplemented with 1% (vol/vol) sodium hypochlorite, and further, both the treated and the untreated suspension were incubated for 24 h at 37°C. Subsequently, both treated and untreated cells were fixed with 2.5% glutaraldehyde (Sigma-Aldrich) in phosphate-buffered saline (PBS; pH 6; Sigma-Aldrich) for 16 h at 4°C. After primary fixation, cells were washed twice with PBS and then fixed with 1% osmium tetroxide for 3 h at 4°C. The fixed cells were dehydrated by using increasing concentrations of ethanol from 30% to 100% followed by drying. The samples (treated and untreated) were further sputter coated with gold (JEC 300 sputter coater) and analyzed under a scanning electron microscope (JEOL JSM 6610LV; Japan) with a ×5,000 magnification.

### Data availability.

The genome sequences of all 25 strains analyzed in the present study are accessible through BioProject number PRJNA889843.
