# Supplementary material for: Genomic Analyses of a Fungemia Outbreak Caused by Lodderomyces elongisporus in a Neonatal Intensive Care Unit in Delhi, India
Source: mBio. 2023 Apr 27;14(3):e00636-23. doi: 10.1128/mbio.00636-23 (PMC10294660; doi:10.1128/mbio.00636-23)
Supplement: FIG S1 [file mbio.00636-23-s0002.docx]

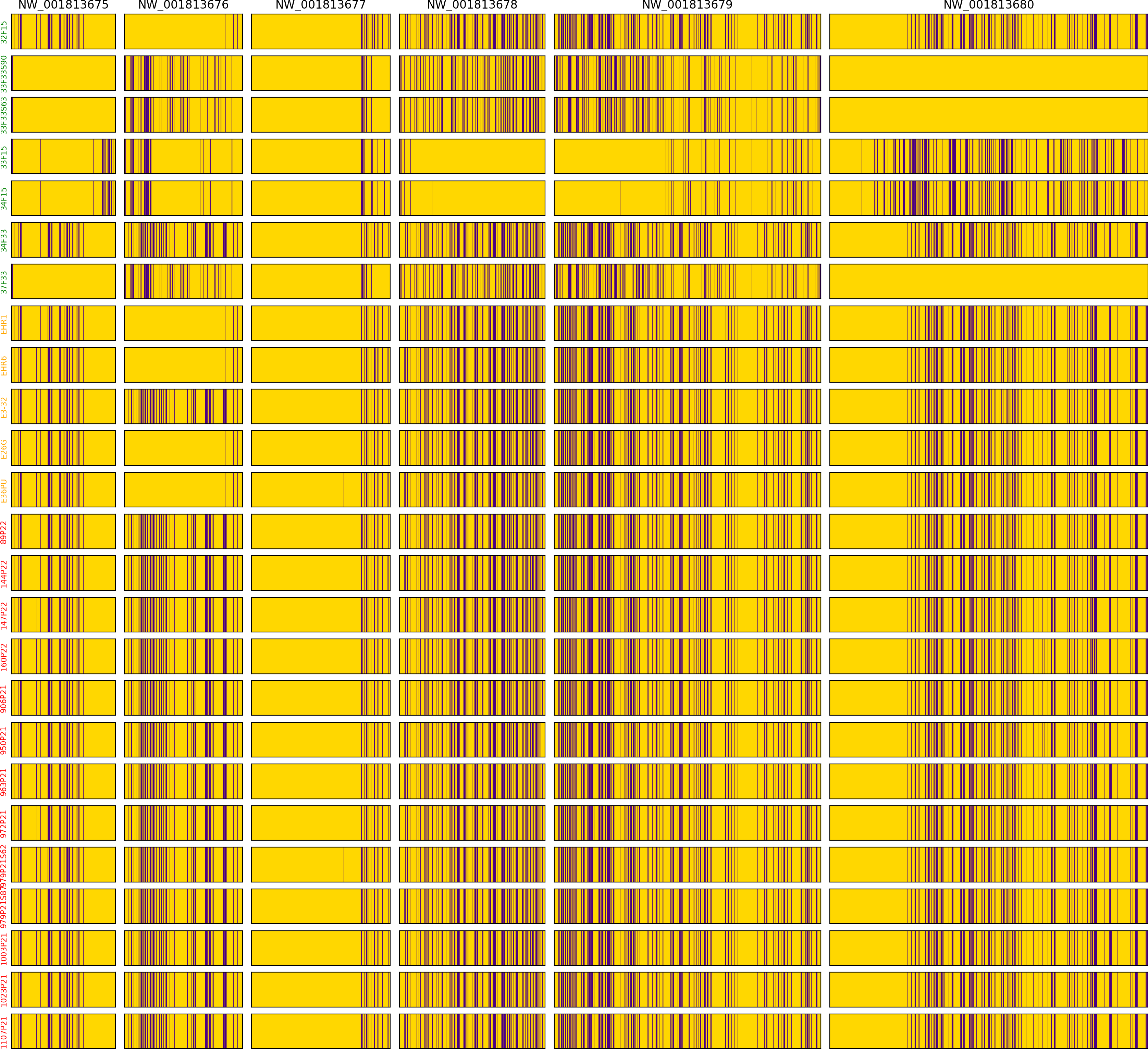


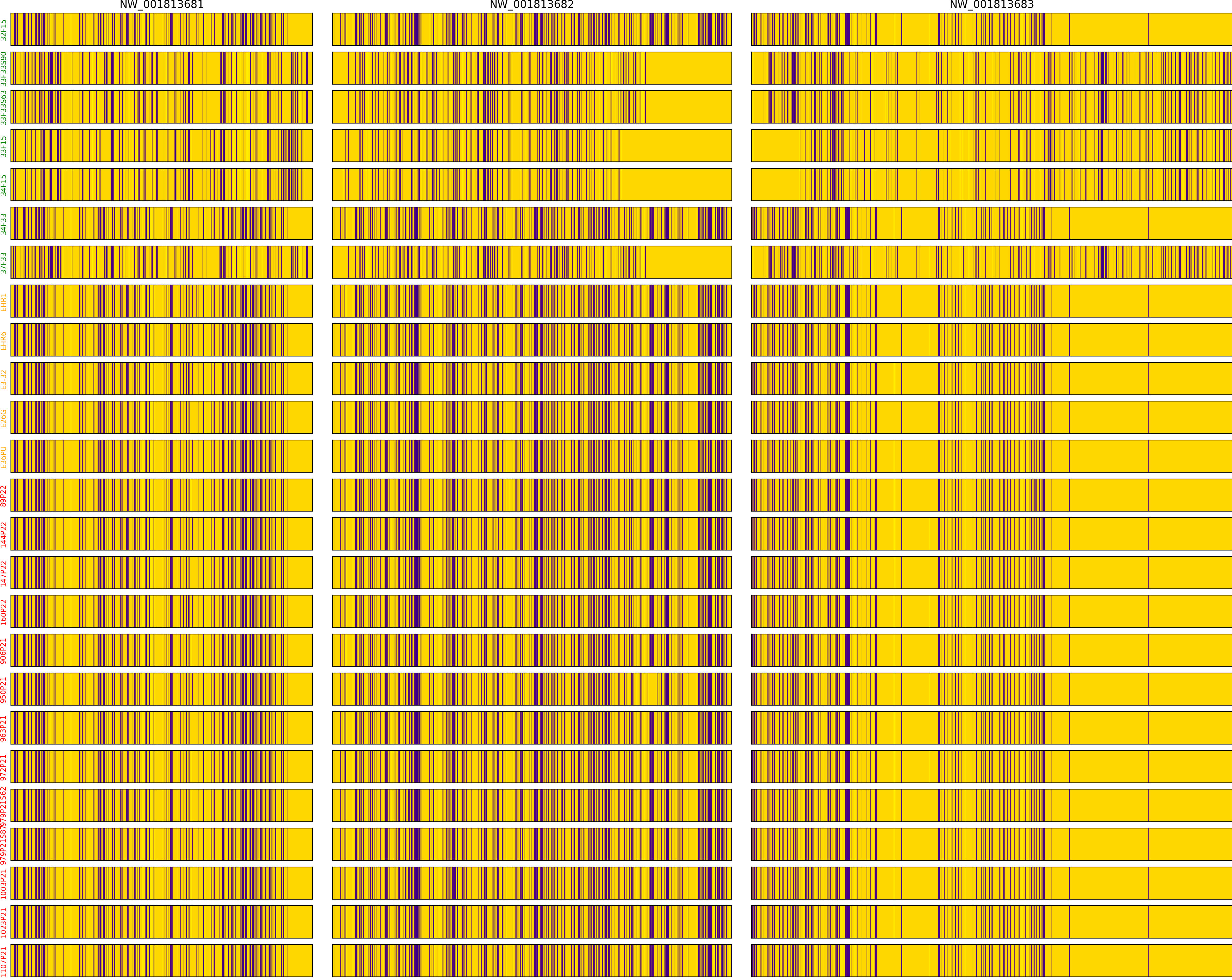


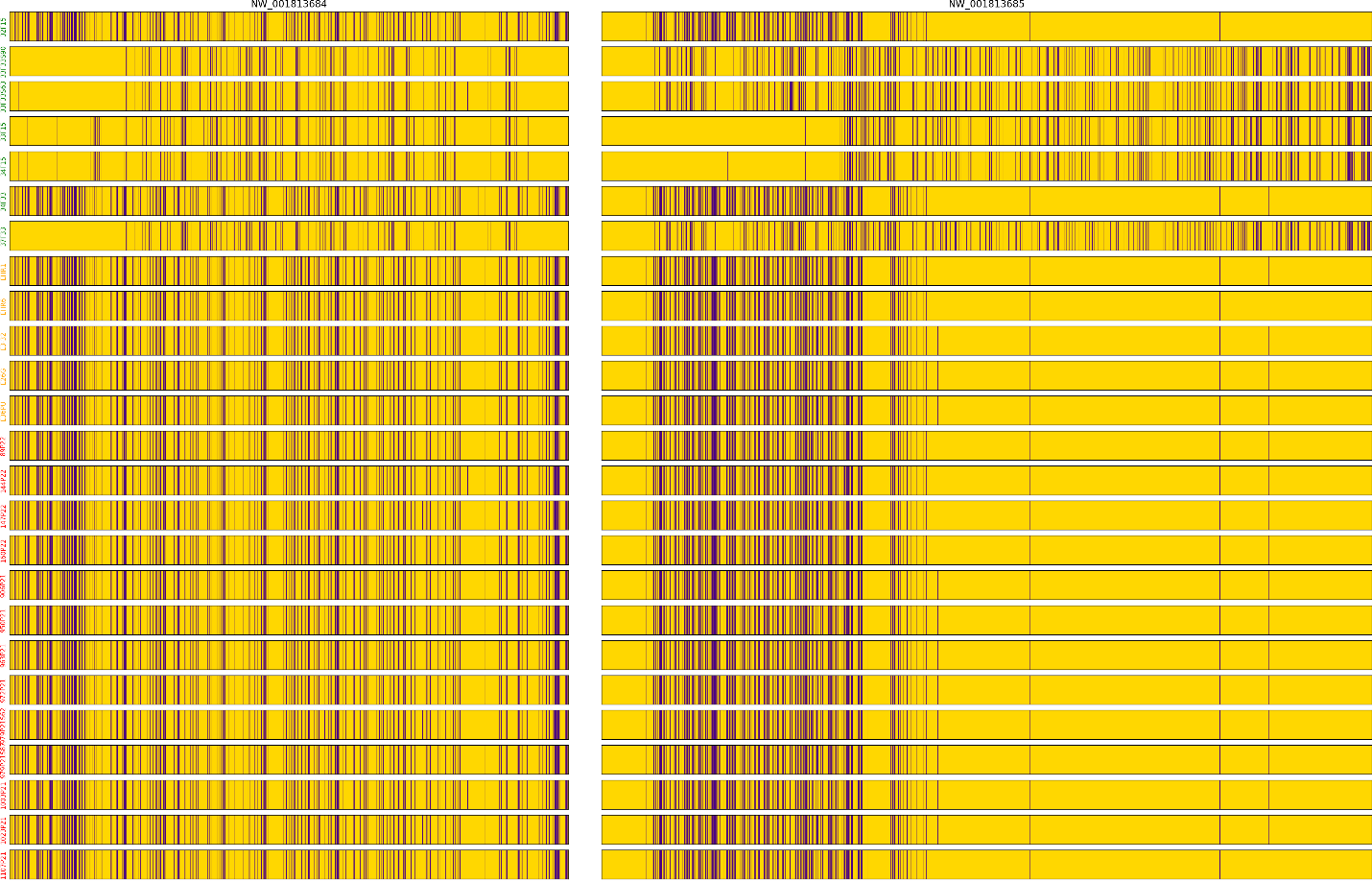


**Figure S1:** Loss of heterozygosity pattern of 25 *Lodderomyces elongisporus* strains including 13 clinical strains and two inanimate environmental strains from the present outbreak and seven strains isolated from the surface of stored apples and three environmental strains obtained from the floor of other two hospitals over the 11 scaffolds. Samples are ordered based on isolation sources. Samples from patients, clinical environment, and fruit were marked by red, orange, and green labels. Purple and yellow blocks indicate heterozygous and homozygous regions.
