# Supplementary material for: Genomic Analyses of a Fungemia Outbreak Caused by Lodderomyces elongisporus in a Neonatal Intensive Care Unit in Delhi, India
Source: mBio. 2023 Apr 27;14(3):e00636-23. doi: 10.1128/mbio.00636-23 (PMC10294660; doi:10.1128/mbio.00636-23)
Supplement: FIG S2 [file mbio.00636-23-s0003.docx]

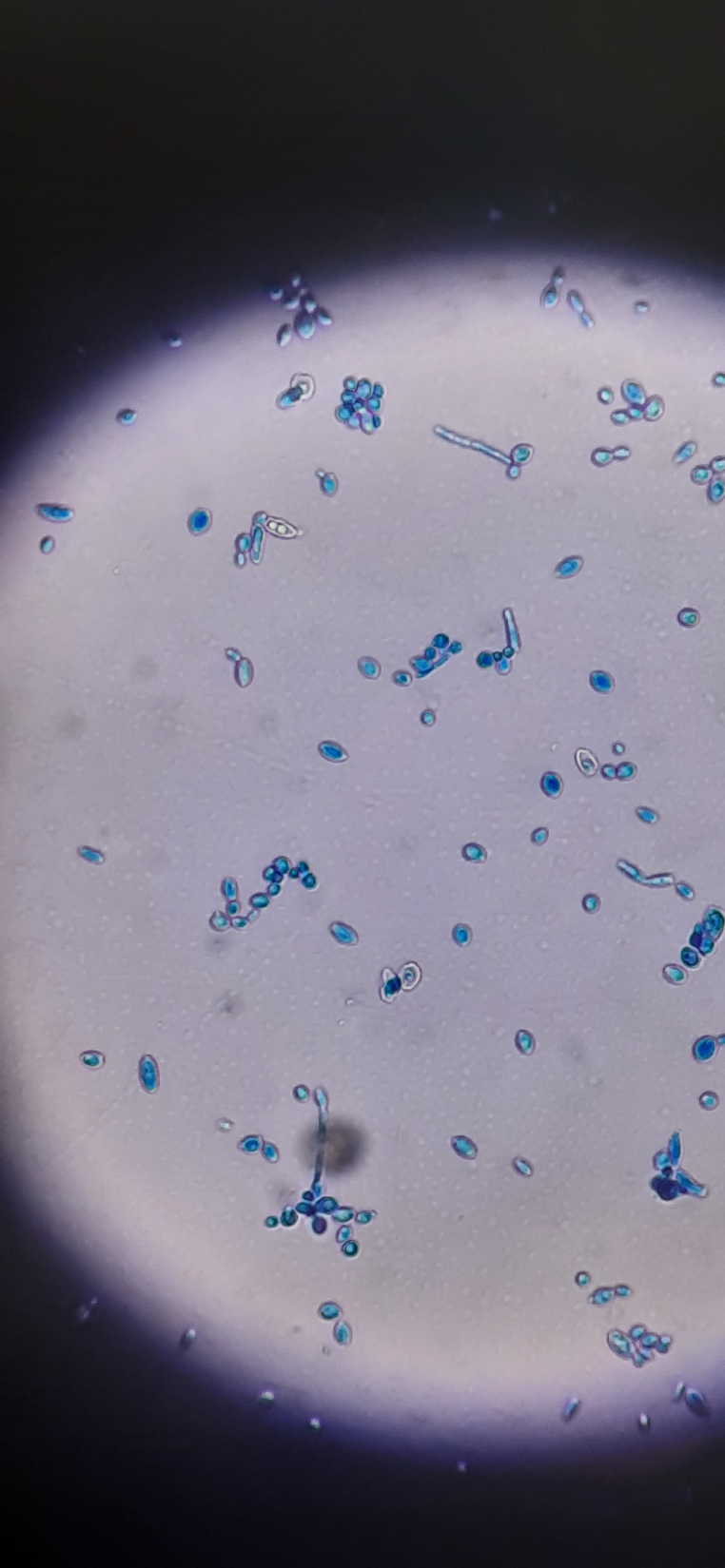


**Figure S2:** Ascospore formation in a clinical isolate (VPCI/160/P/2022) on ascospore agar containing 0.1% sodium acetate stained with lactophenol cotton blue at 40x magnification showing one or two ascospores (white arrow) enclosed in an ascus.
