# Supplementary material for: Genomic Analyses of a Fungemia Outbreak Caused by Lodderomyces elongisporus in a Neonatal Intensive Care Unit in Delhi, India
Source: mBio. 2023 Apr 27;14(3):e00636-23. doi: 10.1128/mbio.00636-23 (PMC10294660; doi:10.1128/mbio.00636-23)
Supplement: FIG S3 [file mbio.00636-23-s0004.docx]

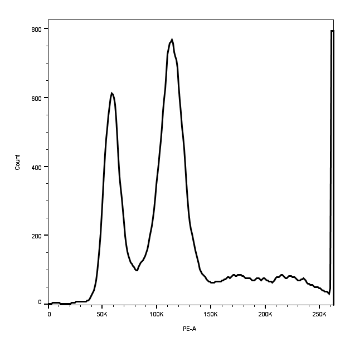


2N

*C. albicans* ATCC 90038


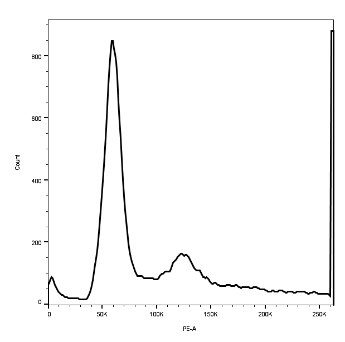

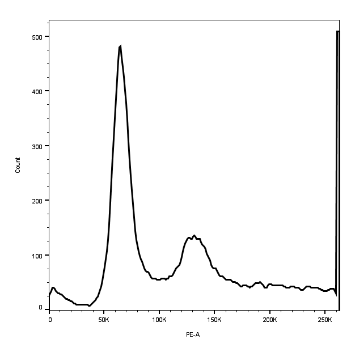


160/P/22 (2N)

147/P/22 (2N)

Clinical strains


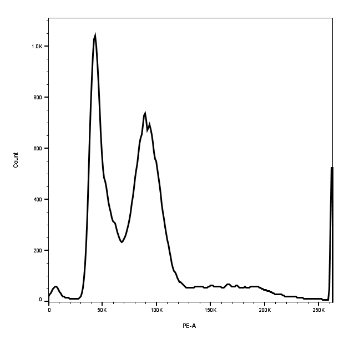

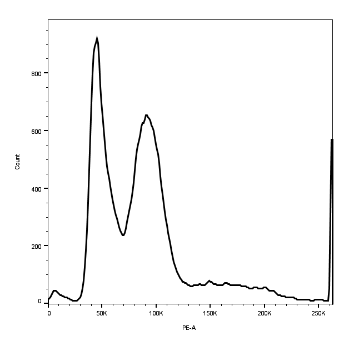


VPCI/E/36PU/2020 (2N)

VPCI/E/28G/2020(2N)

Inanimate environment

from another hospital


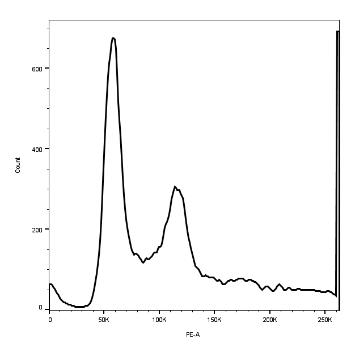


VPCI/E3/32/2020 (2N)


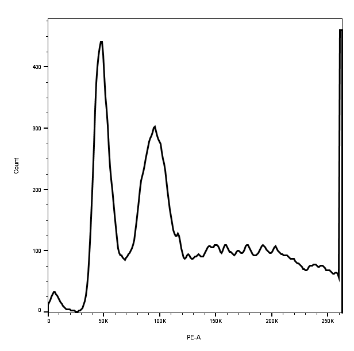

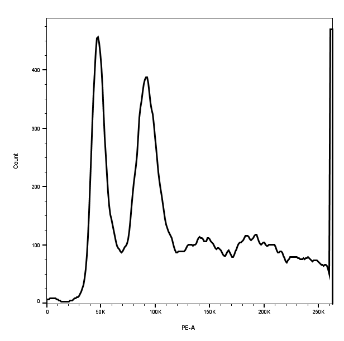


VPCI/33F15/2020 (2N)

VPCI/32F15/2020 (2N)

Fruit strains


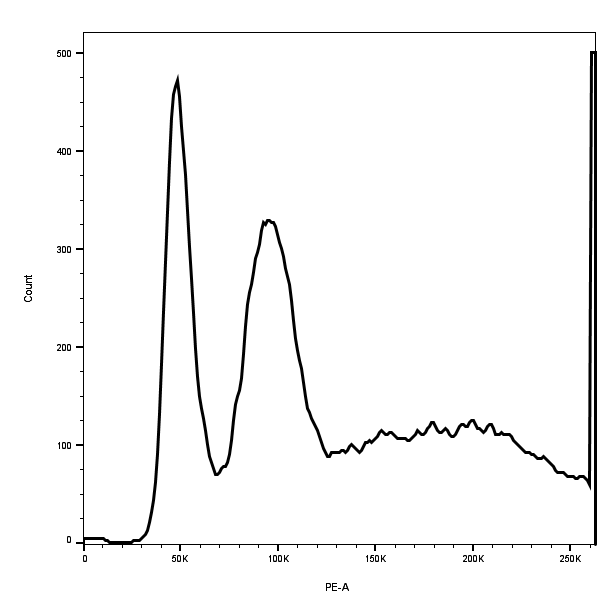

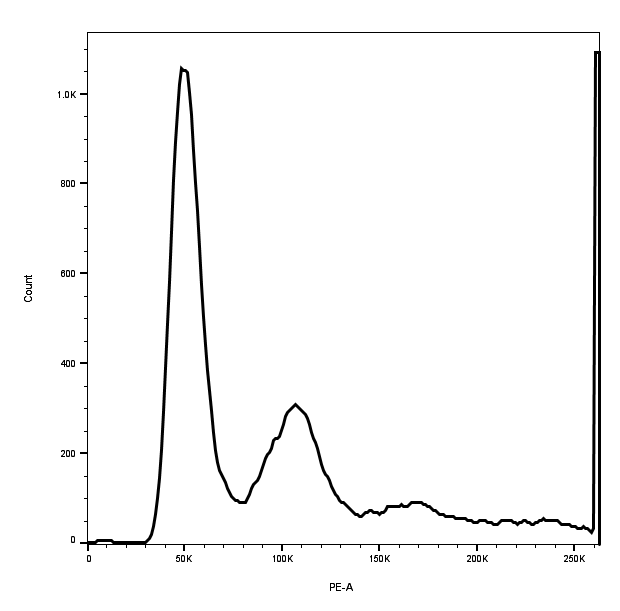


VPCI/33F33/2020 (2N)

VPCI/34F15/2020 (2N)


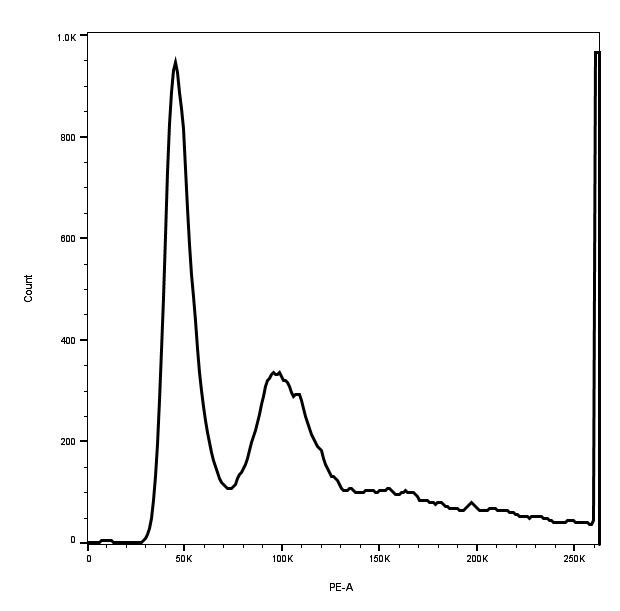

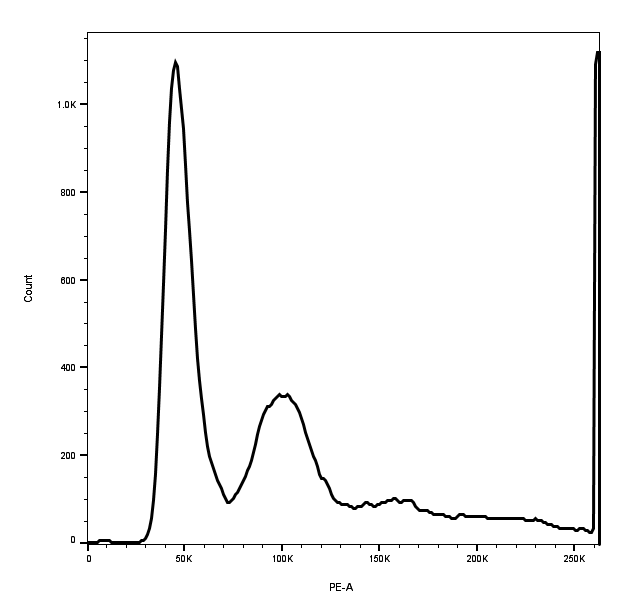


VPCI/34F33/2020 (2N)

VPCI/37F33/2020 (2N)


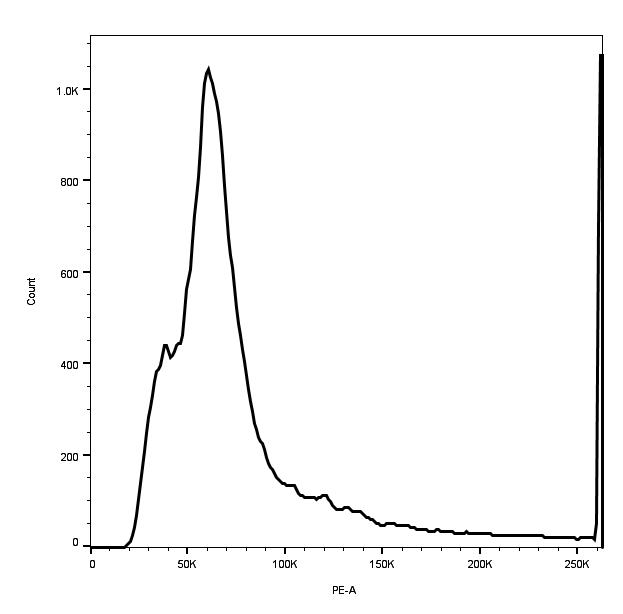

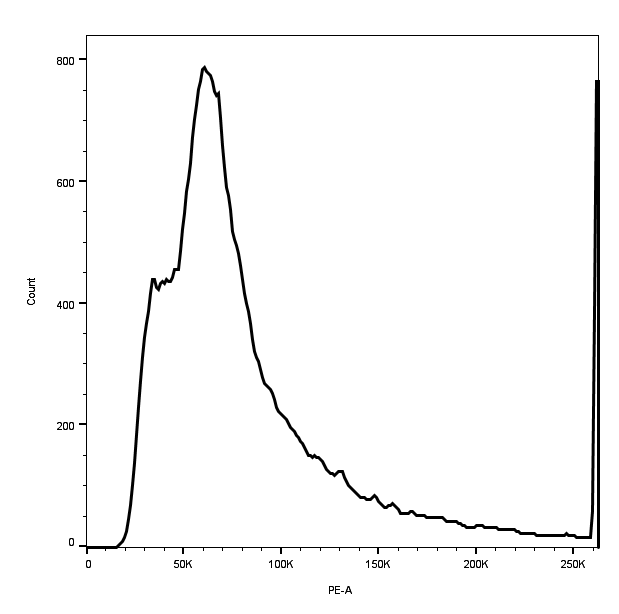


VPCI/E/HR1/22 (aneuploid)

VPCI/E/HR6/22 (aneuploid)

B(

Inanimate environment from present outbreak

**Figure S3:** Histograms representing DNA content or cell cycle profile obtained by FACS. For comparison, a diploid *C.albicans* ATCC90038 was used as reference strain. The x-axis represents nuclear fluorescence, and y-axis represents cell number.
