# Supplementary material for: Genomic Analyses of a Fungemia Outbreak Caused by Lodderomyces elongisporus in a Neonatal Intensive Care Unit in Delhi, India
Source: mBio. 2023 Apr 27;14(3):e00636-23. doi: 10.1128/mbio.00636-23 (PMC10294660; doi:10.1128/mbio.00636-23)
Supplement: FIG S4 [file mbio.00636-23-s0005.docx]

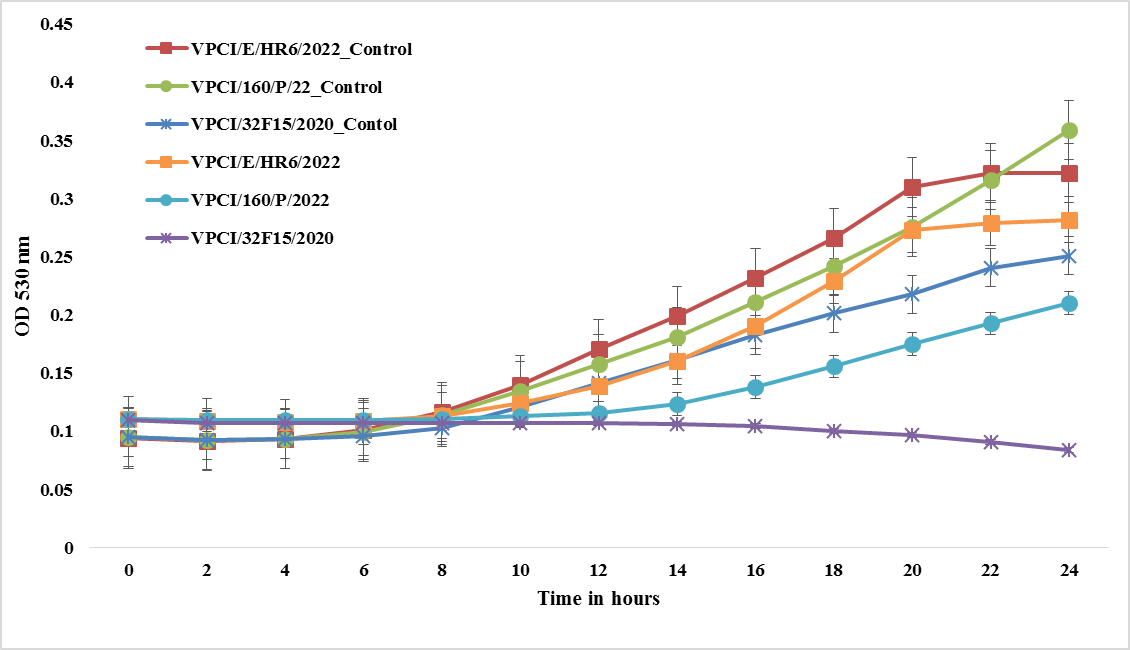


**Figure S4:** The graph illustrates the time-kill curve (optical density versus time plot) of three *Lodderomyces elongisporus* strains from clinical (VPCI/160/P/2022) neonate warmer (inanimate environment VPCI/E/HR6/2022) and from apple surface (VPCI/32F15/2020) in the presence of the 1% (v/v) sodium hypochlorite. The untreated inoculum was used as a control.
