# Supplementary material for: Genomic Analyses of a Fungemia Outbreak Caused by Lodderomyces elongisporus in a Neonatal Intensive Care Unit in Delhi, India
Source: mBio. 2023 Apr 27;14(3):e00636-23. doi: 10.1128/mbio.00636-23 (PMC10294660; doi:10.1128/mbio.00636-23)
Supplement: FIG S5 [file mbio.00636-23-s0006.docx]

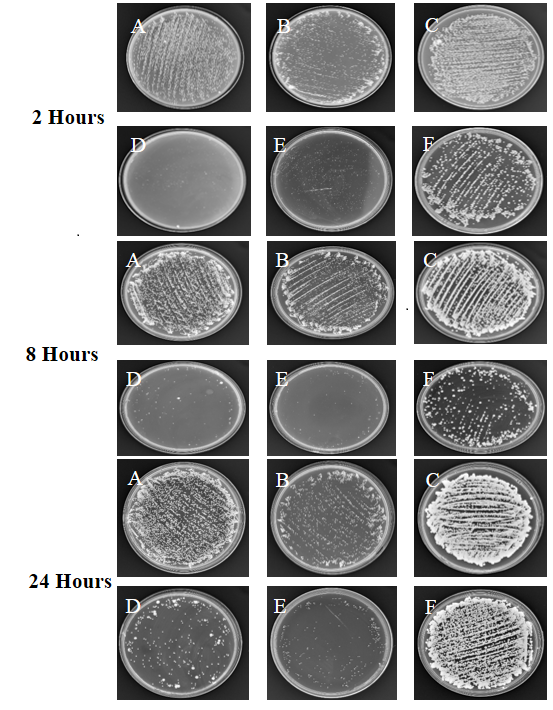


**Figure S5:** Representation of viable cells on culture plates by in use testing in the presence of 1% sodium hypochlorite. Time of incubation is depicted horizontally. A (VPCI/160/P/2022; clinical), B (VPCI/32F15/2020; from apple surface), and C (inanimate environment VPCI/E/HR6/2022; neonate warmer) represents three *Lodderomyces elongisporus* strains in the absence of sodium hypochlorite. Panels D E and F represents the three *L. elongisporus* strains in the presence of 1% sodium hypochlorite in similar order.
